# 1-(4-Methyl­benzyl­ideneamino)pyridinium iodide

**DOI:** 10.1107/S1600536808021569

**Published:** 2008-07-16

**Authors:** Yong-Tao Cui, Chun-Xiang Ji, Wei You, Jia-Sen Sun, Cheng Guo

**Affiliations:** a College of Science, Nanjing University of Technology, Xinmofan Road No. 5, Nanjing 210009, People’s Republic of China

## Abstract

The title compound, C_13_H_13_N_2_^+^·I^−^, is a derivative of 1-amino­pyridinium iodide. The pyridine and benzene rings are oriented at a dihedral angle of 45.78 (3)°. In the crystal structure, weak inter­molecular C—H⋯I hydrogen bonds link the mol­ecules.

## Related literature

For bond-length data, see: Allen *et al.* (1987 ▶).
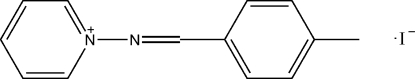


## Experimental

### 

#### Crystal data

C_13_H_13_N_2_^+^·I^−^
*M* *_r_* = 324.15
Orthorhombic, 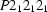

*a* = 7.1690 (14) Å
*b* = 12.399 (3) Å
*c* = 15.026 (3) Å
*V* = 1335.6 (5) Å^3^
*Z* = 4
Mo *K*α radiation
μ = 2.37 mm^−1^
*T* = 291 (2) K
0.30 × 0.10 × 0.10 mm

#### Data collection

Enraf–Nonius CAD-4 diffractometer
Absorption correction: ψ scan (North *et al.*, 1968 ▶) *T*_min_ = 0.536, *T*_max_ = 0.797
1408 measured reflections
1408 independent reflections
1015 reflections with *I* > 2σ(*I*)
3 standard reflections frequency: 120 min intensity decay: none

#### Refinement

*R*[*F* ^2^ > 2σ(*F* ^2^)] = 0.048
*wR*(*F* ^2^) = 0.125
*S* = 1.08
1408 reflections
146 parameters
H-atom parameters constrained
Δρ_max_ = 1.10 e Å^−3^
Δρ_min_ = −0.54 e Å^−3^
Absolute structure: Flack (1983 ▶), with no Friedel pairs
Flack parameter: 0.05 (10)

### 

Data collection: *CAD-4 Software* (Enraf–Nonius, 1989 ▶); cell refinement: *CAD-4 Software*; data reduction: *XCAD4* (Harms & Wocadlo, 1995 ▶); program(s) used to solve structure: *SHELXS97* (Sheldrick, 2008 ▶); program(s) used to refine structure: *SHELXL97* (Sheldrick, 2008 ▶); molecular graphics: *ORTEP-3 for Windows* (Farrugia, 1997 ▶); software used to prepare material for publication: *SHELXTL* (Sheldrick, 2008 ▶).

## Supplementary Material

Crystal structure: contains datablocks global, I. DOI: 10.1107/S1600536808021569/hk2492sup1.cif

Structure factors: contains datablocks I. DOI: 10.1107/S1600536808021569/hk2492Isup2.hkl

Additional supplementary materials:  crystallographic information; 3D view; checkCIF report

## Figures and Tables

**Table 1:** Hydrogen-bond geometry (Å, °)

*D*—H⋯*A*	*D*—H	H⋯*A*	*D*⋯*A*	*D*—H⋯*A*
C9—H9⋯I^i^	0.93	3.06	3.795 (12)	138
C12—H12⋯I^ii^	0.93	3.03	3.929 (13)	162

Symmetry codes: (i) 
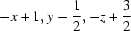
; (ii) 
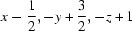
.

**Fig. 1.**
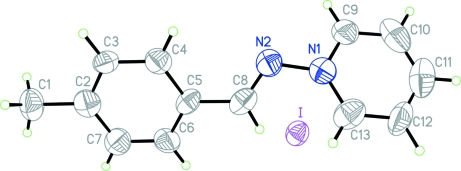
The molecular structure of the title molecule, with the atom-numbering scheme. Displacement ellipsoids are drawn at the 50% probability level

**Fig. 2.**
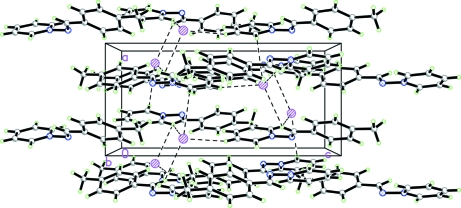
A packing diagram of the title molecule. Hydrogen bonds are shown as dashed lines.

## supplementary crystallographic information

### Comment

Some derivatives of 1-aminopyidinium iodide is important chemical materials. We report herein the crystal structure of the title compound.

In the molecule of the title compound (Fig. 1), the bond lengths (Allen *et al.*,  1987) and angles are within normal ranges. Rings A (C2-C7) and B (N1/C9-C13) are, of course, planar and they are oriented at a dihedral angle of A/B = 45.78 (3)°.

In the crystal structure, weak intermolecular C-H···I hydrogen bonds (Table 1) link the molecules (Fig. 2), in which they may be effective in the stabilization of the structure.

### Experimental

For the preparation of the title compound, 1-aminopyridinium iodide (22.2 g, 0.10 mol) was dissolved in ethanol (20 ml). 4-Methylbenzaldehyde (32.4 g, 0.1 mol) was added with stirring, and then the mixture was heated at reflux for 5 h. Upon cooling to room temperature, a precipitate formed, which was collected by filtration and washed with cold ethanol (2 x 10 ml) to obtain a yellow solid (yield; 38 g, 70%). Crystals suitable for X-ray analysis were obtained by slow evaporation of an ethanol solution.

### Refinement

H atoms were positioned geometrically, with C-H = 0.93 and 0.96 Å for aromatic and methyl H, respectively, and constrained to ride on their parent atoms with U_iso_(H) = xU_eq_(C), where x = 1.5 for methyl H and x = 1.2 for aromatic H atoms.

### Crystal data

**Table:**

C_13_H_13_N_2_^+^·I^–^	*F*_000_ = 632
*M**_r_* = 324.15	*D*_x_ = 1.612 Mg m^−^^3^
Orthorhombic, *P*2_1_2_1_2_1_	Mo *K*α radiation λ = 0.71073 Å
Hall symbol: P 2ac 2ab	Cell parameters from 25 reflections
*a* = 7.1690 (14) Å	θ = 2.1–25.3º
*b* = 12.399 (3) Å	µ = 2.37 mm^−^^1^
*c* = 15.026 (3) Å	*T* = 291 (2) K
*V* = 1335.6 (5) Å^3^	Block, yellow
*Z* = 4	0.30 × 0.10 × 0.10 mm

### Data collection

**Table:**

Enraf–Nonius CAD-4 diffractometer	*R*_int_ = 0.0000
Radiation source: fine-focus sealed tube	θ_max_ = 25.3º
Monochromator: graphite	θ_min_ = 2.1º
*T* = 291(2) K	*h* = 0→8
ω/2θ scans	*k* = 0→14
Absorption correction: ψ scan(North *et al.*, 1968)	*l* = 0→18
*T*_min_ = 0.536, *T*_max_ = 0.797	3 standard reflections
1408 measured reflections	every 120 min
1408 independent reflections	intensity decay: none
1015 reflections with *I* > 2σ(*I*)	

### Refinement

**Table:**

Refinement on *F*^2^	Hydrogen site location: inferred from neighbouring sites
Least-squares matrix: full	H-atom parameters constrained
*R*[*F*^2^ > 2σ(*F*^2^)] = 0.048	*w* = 1/[σ^2^(*F*_o_^2^) + (0.0599*P*)^2^ + 0.581*P*] where *P* = (*F*_o_^2^ + 2*F*_c_^2^)/3
*wR*(*F*^2^) = 0.125	(Δ/σ)_max_ < 0.001
*S* = 1.08	Δρ_max_ = 1.10 e Å^−^^3^
1408 reflections	Δρ_min_ = −0.54 e Å^−^^3^
146 parameters	Extinction correction: none
Primary atom site location: structure-invariant direct methods	Absolute structure: Flack (1983), with no Friedel pairs
Secondary atom site location: difference Fourier map	Flack parameter: 0.05 (10)

### Special details

**Table:**

Geometry. All e.s.d.'s (except the e.s.d. in the dihedral angle between two l.s. planes) are estimated using the full covariance matrix. The cell e.s.d.'s are taken into account individually in the estimation of e.s.d.'s in distances, angles and torsion angles; correlations between e.s.d.'s in cell parameters are only used when they are defined by crystal symmetry. An approximate (isotropic) treatment of cell e.s.d.'s is used for estimating e.s.d.'s involving l.s. planes.
Refinement. Refinement of F^2^ against ALL reflections. The weighted R-factor wR and goodness of fit S are based on F^2^, conventional R-factors R are based on F, with F set to zero for negative F^2^. The threshold expression of F^2^ > 2sigma(F^2^) is used only for calculating R-factors(gt) etc. and is not relevant to the choice of reflections for refinement. R-factors based on F^2^ are statistically about twice as large as those based on F, and R- factors based on ALL data will be even larger.

### Fractional atomic coordinates and isotropic or equivalent isotropic displacement parameters (Å^2^)

**Table:**

	*x*	*y*	*z*	*U*_iso_*/*U*_eq_	
I	0.63765 (9)	0.75120 (8)	0.67606 (4)	0.0632 (3)	
N1	0.1554 (13)	0.6174 (7)	0.7189 (6)	0.049 (2)	
N2	0.1485 (13)	0.6612 (8)	0.8065 (6)	0.051 (2)	
C1	0.241 (2)	1.0008 (11)	1.1391 (8)	0.068 (4)	
H1A	0.3685	1.0169	1.1541	0.102*	
H1B	0.1854	0.9596	1.1862	0.102*	
H1C	0.1735	1.0669	1.1313	0.102*	
C2	0.2358 (15)	0.9358 (10)	1.0529 (7)	0.053 (3)	
C3	0.1465 (17)	0.8349 (9)	1.0519 (7)	0.055 (3)	
H3	0.0964	0.8069	1.1041	0.065*	
C4	0.1329 (14)	0.7771 (8)	0.9731 (7)	0.048 (3)	
H4	0.0746	0.7101	0.9727	0.058*	
C5	0.2057 (14)	0.8187 (9)	0.8955 (7)	0.044 (3)	
C6	0.2865 (17)	0.9199 (8)	0.8960 (8)	0.055 (3)	
H6	0.3312	0.9490	0.8431	0.066*	
C7	0.3023 (16)	0.9792 (10)	0.9746 (8)	0.059 (3)	
H7	0.3570	1.0472	0.9742	0.071*	
C8	0.2037 (13)	0.7578 (10)	0.8115 (6)	0.051 (3)	
H8	0.2447	0.7918	0.7599	0.062*	
C9	0.2109 (17)	0.5139 (9)	0.7163 (9)	0.061 (3)	
H9	0.2361	0.4769	0.7688	0.073*	
C10	0.230 (2)	0.4624 (11)	0.6341 (12)	0.081 (5)	
H10	0.2715	0.3914	0.6305	0.097*	
C11	0.1874 (17)	0.5193 (13)	0.5595 (9)	0.072 (4)	
H11	0.1953	0.4859	0.5042	0.087*	
C12	0.1332 (19)	0.6237 (12)	0.5642 (8)	0.070 (4)	
H12	0.1104	0.6627	0.5125	0.084*	
C13	0.1126 (18)	0.6708 (11)	0.6443 (8)	0.065 (3)	
H13	0.0681	0.7411	0.6478	0.078*	

### Atomic displacement parameters (Å^2^)

**Table:**

	*U*^11^	*U*^22^	*U*^33^	*U*^12^	*U*^13^	*U*^23^
C1	0.078 (10)	0.073 (8)	0.054 (7)	0.006 (8)	−0.006 (8)	−0.019 (7)
C2	0.046 (7)	0.058 (8)	0.055 (7)	0.013 (6)	−0.012 (6)	0.001 (6)
C3	0.068 (8)	0.046 (6)	0.050 (7)	0.009 (7)	0.016 (6)	0.011 (5)
C4	0.052 (6)	0.044 (7)	0.048 (5)	0.002 (5)	−0.007 (5)	0.001 (5)
C5	0.033 (5)	0.060 (7)	0.038 (6)	0.007 (5)	0.006 (5)	0.006 (5)
C6	0.059 (7)	0.043 (6)	0.062 (7)	−0.007 (6)	0.020 (6)	0.001 (6)
C7	0.054 (7)	0.064 (8)	0.059 (7)	0.007 (6)	0.002 (7)	0.000 (7)
C8	0.039 (4)	0.073 (8)	0.042 (5)	0.013 (8)	0.001 (4)	0.011 (8)
C9	0.068 (8)	0.055 (8)	0.061 (7)	−0.011 (7)	−0.013 (7)	0.007 (6)
C10	0.070 (10)	0.064 (9)	0.108 (12)	−0.016 (9)	0.011 (10)	−0.026 (10)
C11	0.046 (8)	0.117 (13)	0.053 (8)	−0.017 (9)	0.015 (6)	−0.025 (9)
C12	0.072 (9)	0.094 (11)	0.044 (7)	−0.016 (9)	−0.018 (7)	0.001 (7)
C13	0.064 (8)	0.075 (8)	0.054 (7)	0.010 (8)	0.007 (7)	0.009 (7)
I	0.0631 (5)	0.0721 (5)	0.0544 (4)	0.0063 (8)	0.0064 (4)	−0.0020 (6)
N1	0.044 (5)	0.050 (5)	0.053 (5)	0.000 (5)	0.006 (5)	0.001 (5)
N2	0.049 (5)	0.054 (5)	0.049 (5)	−0.012 (5)	0.021 (5)	−0.005 (4)

### Geometric parameters (Å, °)

**Table:**

C1—C2	1.526 (15)	C8—N2	1.264 (13)
C1—H1A	0.9600	C8—H8	0.9300
C1—H1B	0.9600	C9—N1	1.344 (13)
C1—H1C	0.9600	C9—C10	1.398 (17)
C2—C7	1.379 (15)	C9—H9	0.9300
C2—C3	1.405 (16)	C10—C11	1.360 (19)
C3—C4	1.387 (15)	C10—H10	0.9300
C3—H3	0.9300	C11—C12	1.353 (17)
C4—C5	1.379 (13)	C11—H11	0.9300
C4—H4	0.9300	C12—C13	1.345 (16)
C5—C6	1.382 (14)	C12—H12	0.9300
C5—C8	1.471 (13)	C13—N1	1.338 (14)
C6—C7	1.396 (15)	C13—H13	0.9300
C6—H6	0.9300	N1—N2	1.425 (11)
C7—H7	0.9300		
			
C2—C1—H1A	109.5	C6—C7—H7	120.4
C2—C1—H1B	109.5	N2—C8—C5	122.7 (9)
H1A—C1—H1B	109.5	N2—C8—H8	118.6
C2—C1—H1C	109.5	C5—C8—H8	118.6
H1A—C1—H1C	109.5	N1—C9—C10	119.4 (12)
H1B—C1—H1C	109.5	N1—C9—H9	120.3
C7—C2—C3	119.7 (11)	C10—C9—H9	120.3
C7—C2—C1	120.6 (12)	C11—C10—C9	118.0 (12)
C3—C2—C1	119.4 (12)	C11—C10—H10	121.0
C4—C3—C2	120.1 (10)	C9—C10—H10	121.0
C4—C3—H3	120.0	C12—C11—C10	121.2 (13)
C2—C3—H3	120.0	C12—C11—H11	119.4
C5—C4—C3	120.2 (10)	C10—C11—H11	119.4
C5—C4—H4	119.9	C13—C12—C11	119.6 (13)
C3—C4—H4	119.9	C13—C12—H12	120.2
C4—C5—C6	119.6 (10)	C11—C12—H12	120.2
C4—C5—C8	122.0 (10)	N1—C13—C12	120.6 (12)
C6—C5—C8	118.4 (10)	N1—C13—H13	119.7
C5—C6—C7	121.1 (11)	C12—C13—H13	119.7
C5—C6—H6	119.4	C13—N1—C9	121.1 (11)
C7—C6—H6	119.4	C13—N1—N2	125.3 (9)
C2—C7—C6	119.2 (11)	C9—N1—N2	113.6 (10)
C2—C7—H7	120.4	C8—N2—N1	113.9 (9)
			
C7—C2—C3—C4	2.9 (16)	N1—C9—C10—C11	−2(2)
C1—C2—C3—C4	177.1 (11)	C9—C10—C11—C12	2(2)
C2—C3—C4—C5	−0.5 (17)	C10—C11—C12—C13	−3(2)
C3—C4—C5—C6	−2.2 (16)	C11—C12—C13—N1	4(2)
C3—C4—C5—C8	176.6 (10)	C12—C13—N1—C9	−3.4 (19)
C4—C5—C6—C7	2.5 (17)	C12—C13—N1—N2	176.5 (11)
C8—C5—C6—C7	−176.3 (10)	C10—C9—N1—C13	2.3 (18)
C3—C2—C7—C6	−2.6 (17)	C10—C9—N1—N2	−177.6 (11)
C1—C2—C7—C6	−176.7 (12)	C5—C8—N2—N1	179.2 (9)
C5—C6—C7—C2	0.0 (18)	C13—N1—N2—C8	−38.8 (15)
C4—C5—C8—N2	−5.4 (16)	C9—N1—N2—C8	141.1 (10)
C6—C5—C8—N2	173.3 (10)		

### Hydrogen-bond geometry (Å, °)

**Table:**

*D*—H···*A*	*D*—H	H···*A*	*D*···*A*	*D*—H···*A*
C9—H9···I^i^	0.93	3.06	3.795 (12)	138
C12—H12···I^ii^	0.93	3.03	3.929 (13)	162

Symmetry codes: (i) −*x*+1, *y*−1/2, −*z*+3/2; (ii) *x*−1/2, −*y*+3/2, −*z*+1.
